# Influence of short-term hypoxic exposure on spatial learning and memory function and brain-derived neurotrophic factor in rats—A practical implication to human's lost way

**DOI:** 10.3389/fnbeh.2024.1330596

**Published:** 2024-02-06

**Authors:** Masataka Kiuchi, Tadashi Uno, Tatsuya Hasegawa, Katsuhiro Koyama, Masahiro Horiuchi

**Affiliations:** ^1^Graduate School Department of Interdisciplinary Research, University of Yamanashi, Kofu, Yamanashi, Japan; ^2^Division of Human Environmental Science, Mount Fuji Research Institute, Fujiyoshida, Yamanashi, Japan; ^3^Faculty of Sport Science, Yamanashi Gakuin University, Kofu, Yamanashi, Japan; ^4^Faculty of Sports and Life Science, National Institute of Fitness and Sports in Kanoya, Kanoya, Kagoshima, Japan

**Keywords:** acclimatization, behavior, hippocampus, normobaric hypoxia, plasticity, simulated high-altitude test

## Abstract

The present study aimed to investigate the effects of a short period of normobaric hypoxic exposure on spatial learning and memory, and brain-derived neurotrophic factor (BDNF) levels in the rat hippocampus. Hypoxic conditions were set at 12.5% O_2_. We compared all variables between normoxic trials (Norm), after 24 h (Hypo-24 h), and after 72 h of hypoxic exposure (Hypo-72 h). Spatial learning and memory were evaluated by using a water-finding task in an open field. Time to find water drinking fountains was significantly extended in Hypo 24 h (36.2 ± 21.9 s) compared to those in Norm (17.9 ± 12.8 s; *P* < 0.05), whereas no statistical differences between Norm and Hypo-72 h (22.7 ± 12.3 s). Moreover, hippocampal BDNF level in Hypo-24 h was significantly lower compared to Norm (189.4 ± 28.4 vs. 224.9 ± 47.7 ng/g wet tissue, *P* < 0.05), whereas no statistically differences in those between Norm and Hypo-72 h (228.1 ± 39.8 ng/g wet tissue). No significant differences in the changes in corticosterone and adrenocorticotropic hormone levels were observed across the three conditions. When data from Hypo-24 h and Hypo-72 h of hypoxia were pooled, there was a marginal negative relationship between the time to find drinking fountains and BDNF (*P* < 0.1), and was a significant negative relationship between the locomotor activities and BDNF (*P* < 0.05). These results suggest that acute hypoxic exposure (24 h) may impair spatial learning and memory; however, it recovered after 72 h of hypoxic exposure. These changes in spatial learning and memory may be associated with changes in the hippocampal BDNF levels in rats.

## 1 Introduction

A recent meta-analysis concluded that hypoxia negatively affects executive functions in humans (McMorris et al., 2017). Specifically, exposure to high altitudes is associated with an impairment of spatial learning and memory (Virues-Ortega et al., 2004). Some studies have shown that short-term acclimatization at high altitudes (days 3–6) improves (recovers) cognitive function impaired by acute exposure to high altitudes (Beidleman et al., 2017; Pun et al., 2018), but not all (Pramsohler et al., 2017; Frost et al., 2021). However, the detailed mechanisms that hypoxic-induced changes in cognitive function has not been well-established. A previous review of human studies summarized that cerebral blood flow (CBF) increases in acute hypoxia (high altitude) to maintain sufficient cerebral oxygen levels (Ainslie and Subudhi, 2014). Despite an increase of global CBF at high altitude, the findings showed that executive function was impaired in hypoxia in human studies (McMorris et al., 2017). In contrast, some animal experiments demonstrated that carotid artery stenosis induced-cerebral hypoperfusion decreased CBF along with cognitive impairment (Gao et al., 2019; Wang et al., 2020). These discrepancies between human and animal findings suggest that global CBF may not fully account for changes in cognitive function, and thus, warrant further investigations. Regional difference in cerebral perfusion (CBF per given volume) related to site specific parts of the brain may be one possible candidate. It was reported that maintenance of appropriate perfusion in the hippocampus is vital to retain memory function (Johnson, 2023). Hippocampal damage may also contribute to cognitive impairment (Gao et al., 2019; Wang et al., 2020).

In the hippocampus of the brain, it has been known that brain derived neurotrophic factor (BDNF) is expressed in high concentration (Hofer et al., 1990; Phillips et al., 1990). BDNF is a member of the neurotrophin family that regulates various neurotrophic functions such as neuroregeneration, neuroprotection, and synaptic plasticity (Mattson et al., 2004). Animal experiments have demonstrated that BDNF plays an important role in spatial learning and memory in rats (Mu et al., 1999; Mizuno et al., 2000; Yamada et al., 2002). Spatial learning and memory function may be important not only in rats but also in humans. For example, 100 million tourists visit high altitudes (e.g., above 2,000 m; Faulhaber et al., 2017). According to the Japanese National Police Agency, ~43% of total numbers of mountain climbing-related accidents were “lose the way,” leading to “fall.” This would be of particular concern to mountaineers for whom falls are serious injury. Since acclimatization at high altitudes would potentially recover hypoxic-induced an impairment of cognitive function (Beidleman et al., 2017; Pun et al., 2018), this raises the question whether changes in spatial learning and memory would be accompanied with BDNF changes during short-term hypoxic exposure.

However, directly evaluating BDNF levels in the hippocampus of an intact human brain is impossible. Alternatively, animal models can allow the evaluation of BDNF levels in the hippocampus though little is known about the time-dependent effects of hypoxic exposure on spatial learning and memory, with respect to changes in hippocampal BDNF levels. Additionally, it has been reported that exposure to hypoxia increased corticosterone and adrenocorticotropic hormone (ACTH) in animal models (Johnson et al., 2013; Wang et al., 2023), which potentially influence cognitive impairment (Reyes-Castro et al., 2018; Lansdell and Dorrance, 2022). Therefore, this study aimed to investigate the effects of short-term normobaric hypoxic exposure on spatial learning and memory. We hypothesized that spatial learning and memory would worsen following acute exposure to hypoxia along with decreases in BDNF levels in the rat hippocampus, but it would recover after short-term acclimatization in hypoxia along with increases in BDNF levels.

## 2 Methods

### 2.1 Animals

All the experiments were performed in accordance with the Ethics Committee for Animal Experiments, Mount Fuji Research Institute, Yamanashi Prefecture Government (ECAE-03-2018E). Forty-five male Wistar rats ranging between 9 and 12 weeks old were used in the experiments. In general, these weeks of age in rats were defined as young adults (Stanley and Shetty, 2004). During the prior training trial, three rats were excluded as they did not move or could not find water fountain within 5-min. Further, during the main experiments, three rats were excluded owing to the same reasons as in the prior training trial. Thus, 39 rats completed one of the three experiments (*n* = 13 for each trial). Ambient temperature (24–26°C) and relative humidity (50–60%) were controlled throughout the experiment. Animals were fed *ad libitum* and kept on a 12 h light–dark cycle.

### 2.2 Experimental protocols

**Figure 1:**
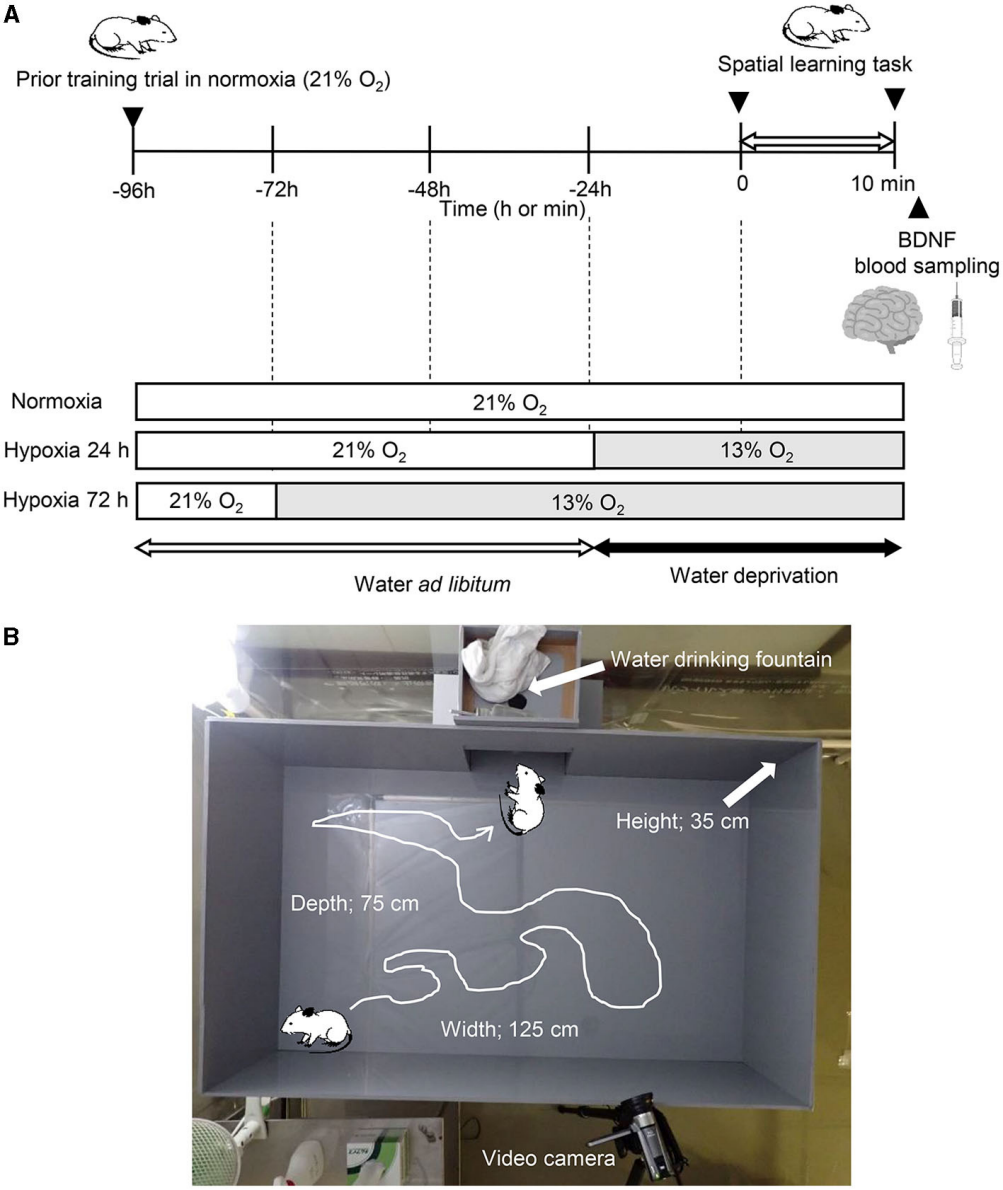
Experimental procedure **(A)** and picture of the apparatus **(B)**.

The protocol consisted of following three conditions of spatial learning and memory tasks in the open field: (1) in normobaric normoxia (room air, 20.9% O_2_, Norm), (2) after 24 h exposure to normobaric hypoxia (12.5% O_2_, equivalent altitude is ~4,000 m, Hypo-24 h), and (3) after 72 h exposure to normobaric hypoxia (12.5% O_2_, Hypo-72 h; Figure 1A). In the two hypoxic trials (Hypo-24 h and Hypo-72 h), hypoxic gas was supplied via a custom-made tent (200 × 200 × 230 cm; width × depth × height) with a hypoxic gas generator system (Hypoxico Everest Summit II: Will Co., Ltd., Tokyo, Japan). The inspired oxygen concentration was determined before and after each experiment (AE-310s; Minato Medical Science, Osaka, Japan). A prior training trial of spatial learning and memory tasks was performed 72 h before each condition. After the spatial learning and memory task (see below) in each main experiment (Norm, Hypo-24 h, and Hypo-72 h), rats were removed from the open field and immediately anesthetized with an anesthetic mixture of medetomidine, midazolam, and butorphanol. Under anesthesia, whole blood samples were collected from the abdominal aorta. After euthanasia due to bleeding from the abdominal aorta, the hippocampi were taken out.

### 2.3 Spatial learning and memory

To assess spatial learning and memory, we used a water-finding task, as described previously (Ichihara et al., 1993; Miyamoto et al., 2001). This test is dependent on latent learning, and selective attention (Cheal, 1980; Ichihara et al., 1993), which has been related to fall risks (Yogev-Seligmann et al., 2008). Briefly, the apparatus consisted of an open field (125 × 75 × 35 cm; width × depth × height) with an alcove (25 × 25 × 20 cm) in the middle of one of the long walls of the enclosure. In the alcove, a drinking tube with the same equipment as usual in the rat's home cage was inserted into the center of the alcove ceiling, with its tip 10 cm above the floor (Figure 1B). During the training task, the rats without water deprivation were placed in one distal corner of the alcove. Then they were allowed to move freely for 5 min. Animals that did not begin exploring within 5 min or did not reach drinking water fountains in the alcoves (*n* = 3) were excluded from the experiments. Rats were immediately returned to the home cage after the training trial and were deprived of water for 24 h before the main experiments (Ichihara et al., 1993; Miyamoto et al., 2001). In the main experiment, the trained rats were placed in the same corner as in the previous training trial of the same apparatus. Two commercial video cameras (OLYMPUS TOUGH TG-6, OLYMPUS Co. Ltd., Tokyo, Japan, and Canon IVIS HF R21, Canon Co. Ltd., Tokyo, Japan) were set 160 cm above the ground of the open field and on the longside wall to the opposite side (Figure 1B). Based on previous studies (Su et al., 2014; Onaolapo et al., 2015), to estimate locomotor activity, the floor (75 cm × 125 cm) was divided equally into 60 squares (i.e., 12.5 × 12.5 cm squares for each) marked by black lines. Total numbers of crossings that the center of body in each rat crossed one side of each grid were counted using the recorded video.

### 2.4 Hippocampal BDNF levels and biomarkers

After learning and memory test, blood sample was drawn from the abdominal aorta, and cardiac arrest was confirmed. Immediately after the euthanasia, the hippocampus was isolated and wet weight of the hippocampus was measured. The hippocampus was completely frozen with liquid nitrogen, and homogenized. A 10 μL phosphate buffered saline, containing 1% protease inhibitor was infused into the hippocampus per 1 mg, and an equivalent ProteoJET™ Mammalian Cell Lysis Reagent (Fermentas, USA) was infused. Thereafter, the sample was centrifuged at 14,000 rpm for 5 min at 4°C (GS-15R, BECKAMAN, Co., Ltd., CA, USA), the supernatant was used to measure the hippocampal BDNF levels using Rat BDNF ELISA kit PicoKine (Boster Biological Technology, CA, USA). Additionally, blood samples were centrifuged at 3,000 rpm for 15 min at 4°C (GS-15R, BECKAMAN, Co., Ltd., CA, USA) to separate serum and plasma, and the plasma was frozen at −80°C for further analysis. Plasma corticosterone and ACTH were analyzed using Corticosterone Multi-Format ELISA Kits (Arbor Assays, Inc., MI, USA) and ACTH (Rat, Mouse)-EIA Kit (Phoenix Pharmaceuticals, Inc., CA, USA), respectively.

### 2.5 Statistics

Data are presented as mean ± standard deviation (SD). All statistical analyses were performed using R software (R ver.3.1.2). One-way analysis of variance (ANOVA) was used to compare all variables among the three different conditions. When a significant *F*-value was found, Dunnett's *post-hoc* test (normoxia data as a control) was performed. To estimate relationships among all outcomes, a *Pearson* correlation coefficient was used. At first, “time to find water fountain” and “total numbers of crossing” were set as dependent variables (i.e., spatial learning and memory function), and hippocampal BDNF levels and plasma biomarkers (ACTH and corticosterone) were set as independent variables. Next, we further sought to investigate relationships between the hippocampal BDNF and plasma biomarkers using a *Pearson* correlation coefficient. In these relationships, we used the data of Hypo-24 h and Hypo-72 h, not the data of Norm to observe hypoxic-induced changes of all outcomes. Effect size (ES) was calculated as Cohen's d where ⩽ 0.2, 0.2, 0.5, and 0.8 were defined as trivial, small, moderate, and large, respectively (Hopkins et al., 2009). Statistical significance was set at *P* < 0.05.

## 3 Results

### 3.1 Body weight

There were no significant differences in rats' body weight among three conditions [262 ± 26 g for normoxia, 281 ± 30 g for Hypo-24 h, and 273 ± 31 g for Hypo-72 h; *F* = 1.44, degrees of freedom (df) = 2 (between), 36 (within), and 38 (total), *P* = 0.257].

### 3.2 Water-finding task

**Figure 2:**
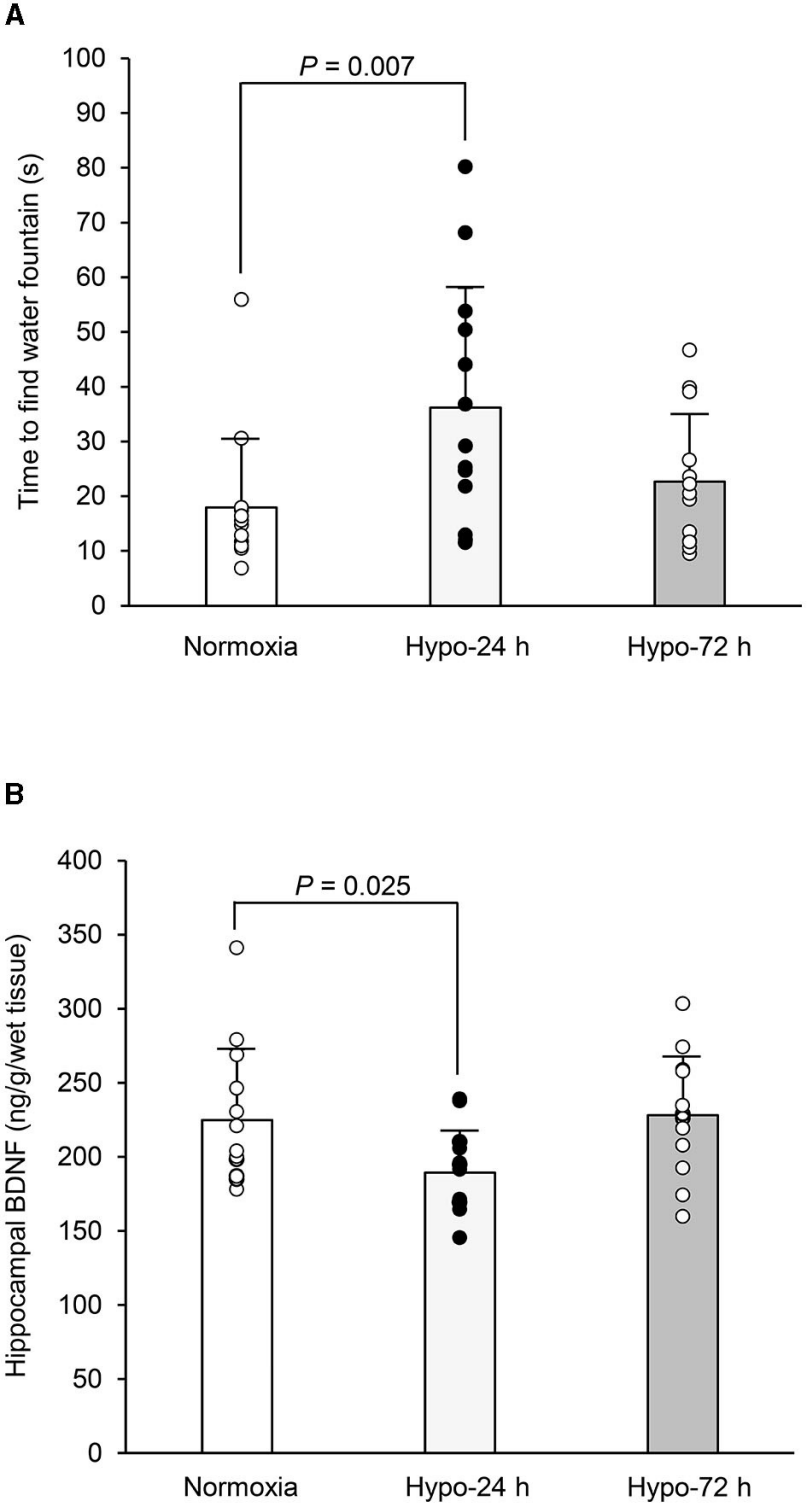
Comparisons in the time to find water drinking fountain **(A)**, and the hippocampal brain-derived neurotrophic factor (BDNF) **(B)** among three trials. The time taken to find drinking water fountains **(A)** and the hippocampal BDNF levels **(B)** in Hypo-24 h were significantly delayed and lower compared to normoxia. Bar graphs indicate mean with standard error bars. Circles indicate individual data (*n* = 13 for each trail). Norm, normoxia; Hypo-24 h, 24 hours exposure to normobaric hypoxia; Hypo-72 h, 72 hours exposure to normobaric hypoxia.

The time taken to find the drinking water fountain under the three conditions is shown in Figure 2A. One-way ANOVA found a significant trial (time) effect (*F* = 4.39, df = 2, 36, and 38, *P* = 0.020, η^2^ = 0.20). Compared to the normoxic trail, the Dunnet *post-hoc* test further revealed that the time to find drinking water fountains in Hypo-24 h was significantly extended (17.9 ± 12.8 s in Norm vs. 36.2 ± 21.9 s in Hypo-24 h, *P* = 0.007), whereas no statistical differences between Norm and Hypo-72 h (22.7 ± 12.3 s, *P* = 0.678) were observed. Locomotor activities in normoxia (15.3 ± 7.6 times) slightly increased in Hypo-24 (19.4 ± 9.6 times), and decreased in Hypo-72 h (13.4 ± 5.1 times) without any statistical differences across the three trials (*F* = 2.08, df =2, 36, and 38, *P* = 0.139, η^2^ = 0.14).

### 3.3 Hippocampal BDNF levels

Figure 2B shows BDNF contents in three trials with a significant trial effect (*F* = 4.39, df = 2, 36, and 38, *P* = 0.020, η^2^ = 0.196). Moreover, compared to the Norm group, BDNF in the Hypo-24 h group was significantly lower (224.9 ± 47.7 in Norm. vs. 189.4 ± 28.4 ng/g wet tissue in Hypo-24 h, *P* = 0.025), whereas no statistical differences between Norm and Hypo-72 h (228.1 ± 39.8 ng/g wet tissue, *P* = 0.744) were observed.

### 3.4 Plasma corticosterone and ACTH

Table 1 shows the changes in the plasma corticosterone and ACTH levels across the three conditions. No significant differences were observed for either variable.

**Table 1:** Changes in corticosterone and ACTH.

	**Normoxia**	**Hypo-24 h**	**Hypo-72 h**	**df**		**F**	***P***
Corticosterone (ng/mL)	1,421 ± 505	1,453 ± 766	1,612 ± 579	Between	2	0.41	0.668
				Within	36		
				Total	38		
ACTH (ng/mL)	2.95 ± 0.78	2.65 ± 0.55	2.75 ± 0.84	Between	2	0.66	0.528
				Within	36		
				Total	38		

Values are mean ± standard deviation.

df, degrees of freedom; ACTH, adrenocorticotropic hormone; Hypo-24, after 24 h exposure to hypoxia; Hypo-72 h, after 72 h exposure to hypoxia.

### 3.5 Relationships among the outcomes

**Figure 3:**
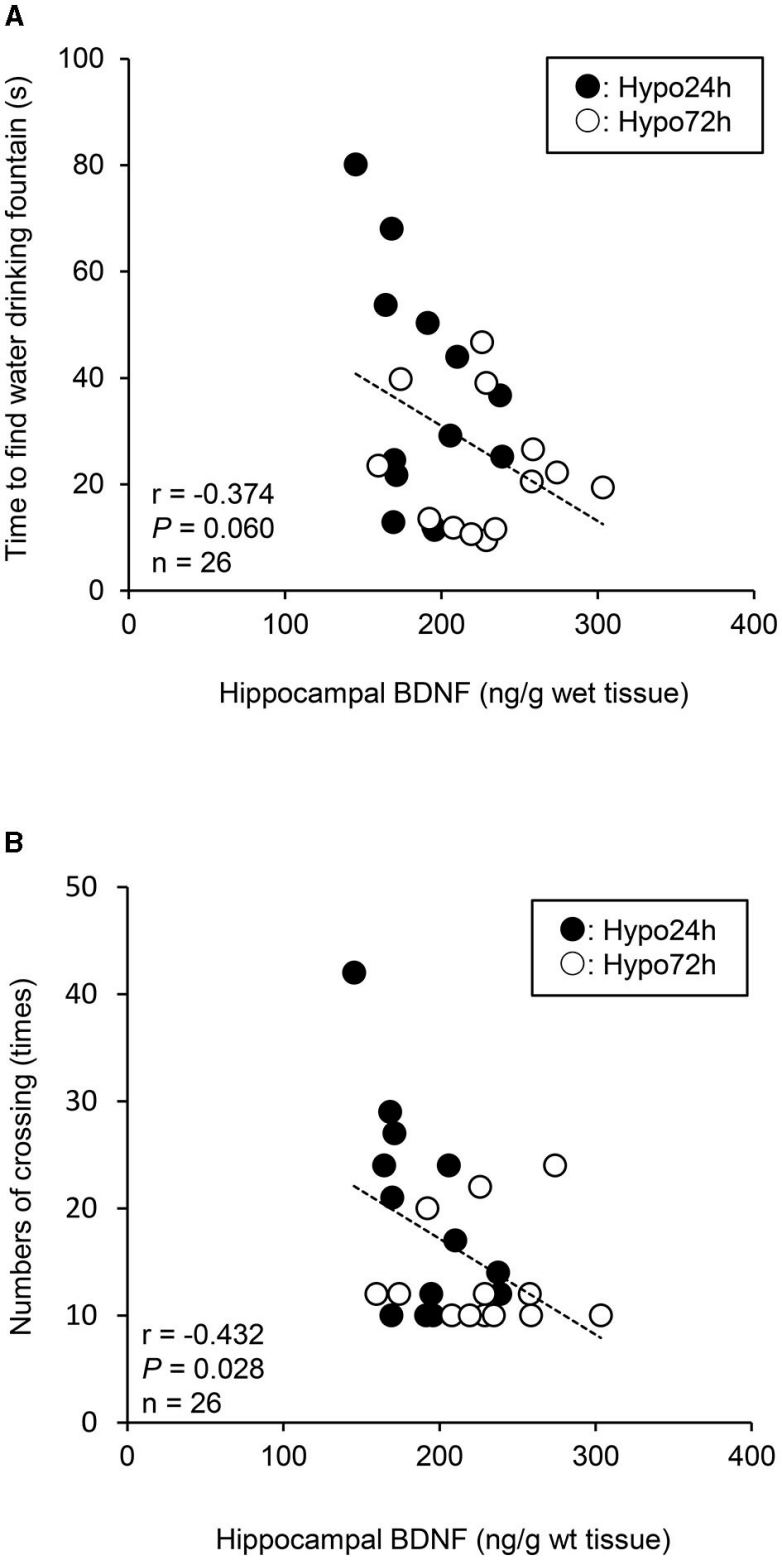
A relationship between the time to find water drinking fountain and hippocampal brain-derived neurotrophic factor (BDNF) **(A)**, and between the total numbers of crossing and hippocampal BDNF **(B)** when the data of Hypo-24 h and −72 h are pooled. Lower BDNF levels caused a longer time to find water fountain, and greater total numbers of crossing in hypoxia. Black and white circles indicate Hypo-24 h (*n* = 13) and 72-h (*n* = 13), respectively.

When the Hypo-24 h and Hypo-72 h data were pooled, there was a marginal relationship between the time to find the drinking water fountain and hippocampal BDNF levels (*P* = 0.060, Figure 3A). Additionally, there was a significant relationship between the total number of crossing and hippocampal BDNF levels (*P* = 0.028, Figure 3B). In contrast, spatial learning and memory function (i.e., time to find the drinking water fountain or hippocampal BDNF levels) was not associated with plasma biomarkers (i.e., ACTH or corticosterone; All *P* > 0.05). Additionally, there were no significant relationships between hippocampal BDNF and ACTH, and between hippocampal BDNF and corticosterone (both *P* > 0.05).

## 4 Discussion

This study is the first to investigate the time-dependent effects of short-term hypoxic exposure on spatial learning and memory, and BDNF levels in the rat hippocampus. The primary findings of the present study were 3-fold: (i) time taken to find water fountain was significantly delayed after 24 h of exposure to hypoxia, but this delay recovered after 72 h; (ii) hippocampal BDNF levels significantly decreased after 24 h of exposure to hypoxia, but to Norm after 72 h; and (iii) when the data of both 24 and 72 h were pooled, a negative marginal trend between time to reach the drinking water fountain and hippocampal BDNF levels was observed.

In agreement with some findings in humans (Beidleman et al., 2017; Pun et al., 2018; Heinrich et al., 2019), our results suggest that short-term acclimatization in hypoxia is a useful tactic to recover (maintain) the same level of spatial learning and memory function as at sea level (in normoxia) after acute impairment of this function. These results provide useful insights for people traveling at high altitudes. Furthermore, previous studies have demonstrated that human serum BDNF levels are associated with cognitive function in humans (Gunstad et al., 2008) and high-altitude exposure (~3,900 m) decreases plasma BDNF levels and impairs cognitive function (Li et al., 2012). Animal experiments in rats found that hippocampal BDNF levels are related to a spatial learning and memory function (Mu et al., 1999; Mizuno et al., 2000; Yamada et al., 2002). Our results showed a negative marginal trend between the time of finding the drinking fountain and hippocampal BDNF levels (*P* = 0.060), and a significant negative relationship between the total numbers of crossing and hippocampal BDNF levels (*P* = 0.028) when both hypoxic data were pooled, suggesting opposite responses between hippocampal BDNF and spatial learning and memory function. Thus, the time-dependent effects of hypoxic exposure on BDNF, spatial learning and memory function could provide further insight than previous human studies (Gunstad et al., 2008; Li et al., 2012). Our findings indicate that hypoxia-induced changes in hippocampal BDNF levels may play a pivotal role in influencing spatial learning and memory during hypoxia. Additionally, the hippocampus is essentially important tissue to maintain spatial learning and memory function (Morris et al., 1982; Sutherland et al., 2001). Moreover, the hippocampus is very vulnerable to insufficient oxygen conditions, such as hypoxia or ischemia (Knierim, 2015), leading to the impairment of memory function even with slight damage (Ocampo et al., 2017). Since BDNF is highly expressed in the hippocampus (Hofer et al., 1990; Phillips et al., 1990), our findings may be supported. However, we must acknowledge that this relationship was observed only under hypoxic conditions, not when normoxic data were included, and the causal relationship between these variables remains unknown.

Unexpectedly, no changes in plasma corticosterone and ACTH levels were observed. Previous animal experiments in rats demonstrated that hypoxic exposure increased corticosterone levels in the plasma (Baitharu et al., 2014) and hippocampus (Baitharu et al., 2012, 2014) along with impaired memory function using the Morris Water maze test. Hypoxia also increases ACTH levels (Raff and Roarty, 1988), which may influence learning and memory in animals and humans (Erickson, 1990). One possibility to account for the inconsistent findings between our study and previous studies (Raff and Roarty, 1988; Baitharu et al., 2012, 2014) may exist. In previous studies, rats were exposed to higher altitudes (25,000 ft = 7,500 m; Baitharu et al., 2012, 2014) with longer periods ~7 days or severe hypoxia (7% O_2_; Raff and Roarty, 1988) than those used in our study. The equivalent altitude of the present study was ~4,000 m. Therefore, although speculative, the lower absolute simulated altitude in the present study may not have been sufficient to increase corticosterone and ACTH levels. Additionally, all animals in the present study were imposed 24-h of water deprivation under all conditions before the main experiment. It has been reported that water restriction increases plasma corticosterone (Arnhold et al., 2009; Izgut-Uysal et al., 2014) and ACTH (Wotus et al., 2007; Arnhold et al., 2009) in rats. Thus, regardless of different oxygen levels (20.9% O_2_ vs. 12.5% O_2_) and different exposure to hypoxia (24 h vs. 72 h), dehydration-induced stress exposure might increase plasma corticosterone and ACTH even under normobaric normoxia condition, resulted in no differences in these variables across the three conditions. Nonetheless, we must acknowledge that this hypothesis is speculative because how hypoxic exposure with water deprivation may further influence water-finding test is unclear.

The detailed mechanisms underlying the impairment of spatial learning and memory after Hypo-24 h recovered at Hypo-72 h, including opposing responses in hippocampal BDNF levels, remain uncertain. The signaling cascades initiated by BDNF and its receptors, such as TrkB are complex (Azman and Zakaria, 2022). The roles of BDNF have been identified, including modulation of neural activity, synaptic transmission, and plasticity (Kowianski et al., 2018), which play important roles in cognitive function (Buchman et al., 2016), learning performance (Deveci et al., 2020) and memory (Hariri et al., 2003; Kambeitz et al., 2012). Moreover, BDNF has been assumed to play a vital role in the plasticity of synapses, including enhancing hippocampal long-term potentiation (Lu et al., 2008) and attenuating long-term depression (Ikegaya et al., 2002), which are considered the primary underlying cellular mechanisms of learning and memory (Cooke and Bliss, 2006). Nonetheless, our experimental design was limited to clarifying these potential mechanisms; therefore, further studies are warranted.

In more details, the present study observed that the hippocampal BDNF levels acutely decreased by 24 h thereafter, increased by 72 h hypoxic exposure. A previous *in vitro* experiment demonstrated that acute exposure to hypoxia (5% O_2_) reduced the expression of BDNF in rat hippocampal neurons and astrocytes in a time-dependent manner (0-1-2-3-6-12 h hypoxic exposure; Tao et al., 2022). Conversely, longer exposure to hypoxia (~10% O_2_) for 72 h stimulated the activation of Wnt/β-catenin signaling using neural stem cells (Qi et al., 2017) or mouse brain (Varela-Nallar et al., 2014), which promotes BDNF expression (Yi et al., 2012; Zhang W. et al., 2018). Thus, the current findings may be supported by these studies; however, we must acknowledge that this hypothesis is highly speculative.

Other potential factors that impair spatial learning and memory should also be considered. For example, animal experiments have shown that exposure to high altitudes impairs memory and increases oxidative stress (Jayalakshmi et al., 2007; Zhang X. Y. et al., 2018). Hypoxia-induced oxidative stress causes rapid impairment of hippocampal mitochondrial biogenesis (Jain et al., 2015), which plays an important role in maintaining cognitive function (Gray et al., 2016; Palomera-Avalos et al., 2017). In contrast, stimulation with an appropriate level of oxidative stress may increase BDNF levels (Siamilis et al., 2009). Thus, how hypoxia-induced oxidative stress affected BDNF levels and spatial learning and memory function in the present study is unknown; however, this point is beyond the scope of the present study.

### 4.1 Methodological considerations

There are some limitations that should be considered to interpret the current findings. First, we used only water-finding task to assess spatial learning and memory. Until now, many tasks such as Water radial-arm maze (Koebele et al., 2019), Morris water maze (Hiroi et al., 2016), and Visible platform (Koebele et al., 2019) tests have been used in this research area. The water-finding task used in the present study is dependent on s dependent on latent learning, and selective attention (Cheal, 1980; Ichihara et al., 1993), and impairments of these function may cause increases in fall risks (Yogev-Seligmann et al., 2008); however, future studies using various tasks could yield further insights about hypoxic exposure-induced changes in spatial learning and memory. Secondly, the sample size was relatively small. However, as no studies have examined the effects of short-term hypoxic exposure on spatial learning and memory and hippocampal BDNF levels, we conducted a *post-hoc* power analysis for pairwise comparisons. We performed the test for variables that were observed with significant differences as the standard of 80% power with a two-sided significance level of 0.05 (G Power 3.1). As a result, the estimated effect size of *Cohen's d* was 1.13 and 0.93, with an actual (1-β) power of 0.821 and 0.818 for time to find a drinking water fountain and hippocampal BDNF levels between Norm and Hypo-24 h. Although future studies with a larger sample may be required to confirm our findings, these numbers may be sufficient to detect significant differences in these variables (Hopkins et al., 2009).

### 4.2 Implications

Compared to previous human studies (Das et al., 2018; Chroboczek et al., 2022) and animal experiments (Mu et al., 1999; Mizuno et al., 2000), the present study has some strengths that can be applied to human's life. For example, human studies could measure only serum BDNF levels owing to be technically impossible for evaluation hippocampal BDNF levels in intact humans (Das et al., 2018; Chroboczek et al., 2022). Moreover, animal experiments used surgically BDNF treated models (Mu et al., 1999; Mizuno et al., 2000), indicating the model is far from humans' life. In this regard, our experimental model can be considered to mimic mountaineering. Our findings may be informative for populations that climb high altitudes, such as Mount Fuji (3,776 m). Specifically, acute exposure to high altitude (i.e., ~24 h) could potentially impair learning and memory function; thus, we propose precautionary measures to reduce loss in the mountains.

## 5 Conclusion

In summary, the current findings suggest that acute hypoxic exposure (24 h) may impair spatial learning and memory; however, short-term hypoxic acclimatization may recover these impairments. Moreover, changes in spatial learning and memory may be explained by changes in the hippocampal BDNF levels in rats.

## Data availability statement

The original contributions presented in the study are included in the article/supplementary material, further inquiries can be directed to the corresponding author.

## Ethics statement

The animal studies were approved by Ethical Committee at Mount Fuji Research Institute. The studies were conducted in accordance with the local legislation and institutional requirements. Written informed consent was not obtained from the owners for the participation of their animals in this study because experimental animals were obtained from commercial company.

## Author contributions

MK: Conceptualization, Data curation, Formal analysis, Investigation, Methodology, Writing—original draft. TU: Conceptualization, Formal analysis, Writing—review & editing, Data curation, Funding acquisition, Investigation. TH: Conceptualization, Formal analysis, Investigation, Writing—review & editing, Methodology. KK: Conceptualization, Methodology, Writing—review & editing, Supervision. MH: Conceptualization, Methodology, Supervision, Writing—review & editing, Formal analysis, Writing— original draft.
